# Family socioeconomic status and the parent–child relationship in Chinese adolescents: the multiple serial mediating roles of visual art activities

**DOI:** 10.1186/s12889-022-13215-8

**Published:** 2022-05-20

**Authors:** Chunhai Gao, Endale Tadesse, Sabika Khalid

**Affiliations:** 1 Faculty of Education, Shenzhen University, Shenzhen, China; 2 Faculty of Education, Southwest University, Educational Leadership and Management, No.2 Tiansheng Road, Beibei District, Chongqing, 400715 PR China

**Keywords:** Visual art activities, Extracurricular activities, Socioeconomic status, Parent–child relationship

## Abstract

**Background:**

In light of the recent policy reform in China, the present study aims to investigate the potential impact of family SES on the quality of the parent–child relationship (PCR) through the serial mediating role of participation in organized visual art activities in privately owned centers (VAA1) and parent-supervised visual art activities (VAA2) across genders.

**Method:**

A cross-sectional study was conducted in anonymous province located in the southwestern part of China. A total of 1624 primary school students aged 7 to 14 years were recruited through a random sampling technique. Subsequently, anonymous survey responses were taken from all students. Multiple serial mediation analysis was performed by using AMOS 21.0 software to attain the primary aim of the study.

**Result:**

According to the total sample model result, SES has a significant direct effect on the parent–child relationship (β = 0.47, *p* < 0.001), children’s participation in VAA1 (β = 0.197, *p* < 0.001) and VAA2 (β = 0.269, *p* < 0.001). Moreover, the mediation model result indicates that SES has a stronger indirect effect on the parent–child relationship through a mediating role of VAA1 (β_girl_ = 0.08, *p* < 0.01; β_boys_ = 0.04, *p* < 0.01) for female than male samples. However, the mediating effect of VVA2 between SES and the parent–child relationship in the female (β = 0.08, *p* < 0.001) and male (β = 0.08, *p* < 0.01) models is equal, although female gender is highly significant. Ultimately, the serial mediation analysis result affirms that the serially mediating role of VAA1 and VAA2 between SES and the parent–child relationship was equal across genders (β_boys_ = 0.001, *p* < 0.001; β_girls_ = 0.001, *p* < 0.001).

**Conclusion:**

Unlike previous studies, this study’s multigroup model shows that both male and female children can equally restore their relationship with their parents by having substantial participation in both VAA1 and VAA2. Thus, parents ought to play the main role in facilitating and supporting children’s visual art activities without parenting that shows a gender bias.

## Introduction

The relationship that children and parents possess is a profound means of restoring or promoting children’s development by providing a sense of security [1–8] and predicting future professional, academic and social achievements [9–12]. Likewise, a large volume of literature pronounced that the quality of the parent–child relationship defines children’s academic achievement [3, 13, 14], mental and emotional well-being [5, 11, 14–18], social competency [19, 20], creativity [21], and behavioral problems [15]. Consequently, numerous scholars have put forth implications that it is important to foster the quality of the parent–child relationship so that children grow in a sustainable environment by creating harmonious interactions with their parents, peers, and community members [18, 20, 22]. The primary suggestion that the literature has made is for parents to improve their involvement in their children's academic, social, and cultural capital development (e.g., [3, 5, 12, 14]). However, the nature of the parent and child relationship is notably affected by cultural and social adjustment [1, 6, 22, 23].

Thus, the present study seeks to investigate the essence of parental involvement from the perspective of visual art activities in restoring the parent–child relationship in China, in light of the recent Chinese government policy reform that targets children and parents. For decades, Chinese parents put extensive pressure on their children’s academic performance through intensive private tutoring classes after primary school time, which led to an adverse effect on children's academic, emotional, and, most importantly, the quality of the parent–child relationship [14]. On the other hand, shared extracurricular activities (ECAs) enhance the emotional attachment between parents and children; explicitly unlike other ECAs, visual art activities such as painting, drawing, sculpture, photography, and crafts do not require deep knowledge and skill from parents in order for them to be involved in, support, and celebrate their children’s creativity [8, 22, 24–28]. Hence, the recent national policy reform is expected to stimulate the traditional parent–child relationship by preventing parents from sending their children to after school tutoring centers and reducing the overwhelming amount of homework that was previously given by teachers so that parents and children can have family time by encouraging parents to become involved in in- or out-of-home ECAs and private owned training centers that service several ECAs [14]. A subsequent national survey study asserts that investing in children's academic learning materials only ensures cognitive development while harming children's intrinsic and emotional well-being [23]. Thus, this study implies that in addition to the viable academic investment, children would benefit from the involvement of parents in activities at home, extracurricular activities, and parent–child communication [23].

Although the recent national policy reform neutralizes the academic-centered parenting style, which does not prioritize emotional attachment among parents and children, the power of socioeconomic status (SES) has been pronounced due to the country's rapid economic development. Accordingly, such social change led parents to be inclined to Western culture, which intensely transformed the recent Chinese parent–child relationship [1, 6], resulting in a conflict between modern and traditional parenting styles [19] that deserves further understanding through research [29]. In addition, it is well documented that in traditional Chinese parenting, which still exists, parents show deeply embedded beliefs regarding the preference of their children’s gender, which determines the intensity of the devotion parents provide to their child in terms of money and time (e.g., [1, 6, 19, 29, 30]). Therefore, shining light on the contemporary Chinese parent–child relationship to the current policy reform, which is speculated to revive the power of SES, is significant. Moreover, a great deal of studies in China that discussed different ECAs, including visual art activities, considered cognitive skills as their dependent variables (e.g., [31, 32]). Hence, this study aims to address the application of visual art activities in promoting the parent–child relationship, which is one of the underexplored consequences of ECAs and assures the well-being of children by weakening the impact of SES [1].

## Literature review

### Socioeconomic status and the parent–child relationship

SES has been associated with the essential factor that determines children’s psychological, mental, academic, and social well-being (e.g., [12, 17, 21, 23, 33–35]). According to Bronfenbrenner's ecological theory, parents' SES level is crucial in the microsystem (Bronfenbrenner, 1979), which has a multidimensional impact on children’s development [7, 10, 12, 13, 15, 20, 32]. For instance, a growing body of evidence elucidates that SES influences children’s mental and psychological health (e.g., [5, 36]), social creativity [20, 21], and academic adjustment [13, 35]; given that educated parents have a higher likelihood of recognizing the significance of children’s psychological, mental, physical, academic and social stability, their children are more likely to access relevant resources that maintain those aspects [7, 12, 35, 37–39]. Nevertheless, a later path analysis study claimed that SES has no direct effect on children's psychological well-being, although it has a robust indirect effect through the mediating role of the parent–child relationship and parental involvement [17]. Simultaneously, based on a study that administers the mediating role of parent–child relations, it significantly accounted for the relationship between SES and academic achievement [13, 35] and children’s internet use [7]. This evidence explains that parents' financial investment in the interest of their children's overall well-being might be ineffective unless parents sustain their relationship and involvement with their children.

Furthermore, an extensive amount of literature has noted that high-SES children possess higher quality peer and parent–child relationships than their low-SES counterparts [1, 5, 12, 13, 17, 29, 35, 36, 40]. Children who secure a quality relationship with their parents build a solid personal identity that allows them to develop genuine social communication skills and learn how to tackle any challenges they face [1, 6, 20, 21]. In contrast, the Family Stress Model [41] states that low-SES children grow up in an environment that neglects their primary needs and is fraught with family conflict, which jeopardizes and aggravates the parent–child relationship and leads to dissatisfied, underperforming, children who have low self-esteem and psychotic tendencies [4, 7, 21, 35, 36, 40]. One recent work is an ideal mediating study that affirmed that the parent–child relationship has a more robust mediating role than the teacher-and-peer relationship between the association between SES and children’s mental health [36]. Following the Family Stress Model, the family investment model [42, 43] proposes a means for parents to enhance their investment in the children (basic needs, learning resources, self-development activities, etc.) to provide health and peaceful living standards for children, leading children to acquire skills that allow academic and social achievement [12, 21, 23, 40]. Emerging evidence in China illustrates that parents’ SES, particularly maternal, [35, 40] and both parents’ education attainment [12, 38], play a substantial role in predicting both mother–child and father-child relationships. In addition, Li et al. study extends the family investment model by presenting the substantial mediating effect of parent–child literacy activities in promoting a positive parent–child relationship [12]. Thus, the present study aims to examine the potential investment (time and money) of contemporary Chinese parents to restore the parent–child relationship through one of the ECAs known for fostering interaction between parents and children [6, 23, 44].

### The mediating role of visual art activities between SES and parent–child relationships

For decades, while SES has shown a robust and powerful predicting power on children’s development outcomes, its effect is mediated by more proximal characteristics of a child’s living environment, which are more directly linked to child development [23]. In- and out-of-school ECAs are known learning environments for expanding children's cognitive and noncognitive skills [45, 46]. However, due to the lack of resources, qualified teachers, and attention from the government, in-school ECAs cannot meet the needs of students, teachers, and parents to achieve their intended goals [47–50]. Subsequently, studies focus on assessing the consequences of different out-of-school ECAs in enhancing children's development (e.g., [32]). The parent–child relationship, which is related to childhood development, cannot occur in a vacuum; it requires a dynamic environment in which parents and children can interact without limits [4, 9, 18, 51]. This makes the parent–child relationship a kind of parental resource because it is closely related to parental time (attention) spent on children or parent–child interactions [6, 7]. Plausibly, although there is a limited number of studies regarding visual art experiences outside of school [10, 46, 52, 53], it is well documented as the most promising ECA known for advocating children’s well-being [2, 22, 24, 25, 51, 54]. Visual art activities are a form of events that children and parents engage in, such as painting, drawing, sculpture, photography, and crafts [8, 16, 18, 22, 24–28, 52, 53]. Despite the fact that these visual art activities broaden children’s horizons to help them view their surroundings and the world differently [9, 18, 26, 51], most evidence has concentrated on determining children’s cognitive development (e.g., [24, 32, 34, 50, 54]). A recent systematic review that synthesizes the literature demonstrates that an overwhelming amount of evidence assesses the relationship between art activities and academic achievement [49], which neglects other vital aspects of children’s development. Additionally, visual art activity participation was measured as an experience at home, in art galleries, community centers, and museums supervised by an adult (parent or caregivers) [10, 28, 33, 45, 55, 56]. Thus, the present study sought to categorize out-of-school visual art activities (a) organized by private training centers and (b) in- and out-of-home visual art activities supervised by parents.

Furthermore, a growing body of literature has indicated that in addition to the positive cognitive development of children, visual art activities have the potential power to stimulate the quality of parent–child communication and relationships [4, 8, 16, 18, 25, 55, 56]. The use of art-based tools during early and middle childhood, in particular, provides a prospective way to learn the nature of the parent–child relationship [9]. A shared activity that involves parents and children can improve the parent–child relationship by helping parents to understand their children’s mindset, given that the activities require intensive communication, collaboration, and support from both parties [2, 8, 9, 18, 22, 25, 33, 34, 52]. Moreover, parents who are involved in and support the home art activities of their children are more likely to become involved in further activities that expand their children's visual art activities [46, 54]. Likewise, children who have a habit of drawing or painting have a higher likelihood of exhibiting proper discipline and avoiding any psychological ill-being [16]. Due to the robust implications of studies concerning the application of visual art activities, they became the primary means of intervention or therapy among psychologists and psychiatrists to support the parent–child relationship (e.g., [2, 4, 8, 9, 18, 55, 56]).

Most interestingly, studies have revealed that low-SES children engaged in visual art activities improve any emotional problems they face due to poverty [16, 50, 51, 55, 56]. Although visual activities benefit disadvantaged children, the possibility of children participating in visual art activities in and out of the home is determined by the SES of parents [10, 22, 32, 46, 53, 54], particularly maternal social position [33, 45]. Unlike Western countries [26–28, 46, 47, 50, 55, 56], in China, intensive organized visual art activities for primary and middle school children are available at private-owned centers and in-home and out-of-home visual art activities supervised by parents, which costs a fortune. Educated and financially capable parents would send their children to private art classes and accompany them to their sessions [32, 38]. Thus, private art centers motivate and stimulate children to develop their self-efficacy by developing peer communication, since visual art activities require intensive interaction among teachers and peers, and allow parents to develop relationships with their children by commenting on their artwork, listening to their experience, and tracking their progress [38], which nurtures parental support by involving in a shared visual art activity in-home and out of home [39]. Providentially, visual activities in early adolescence do not need deep knowledge and understanding for parents to provide support, in contrast to music, sports, or other ECAs that require factual information for parents to provide appropriate support or give constructive comments.

Hence, affluent children show receptive visual art participation in paid art classes; they have a higher probability of enjoying visual art activities at home and attending art galleries, visiting museums, and meeting iconic artists due to their parental cultural capital [34, 38, 47, 53]. Consequently, affluent children participate in organized visual art activities in private centers and adult-supervised visual art activities in and out of the home, which reinforces social inequality [53, 54] and shows the importance of cultural capital [28, 33, 34, 39, 47, 53]. In contrast, low-SES children spend their leisure time on activities that are unsupervised by adults such as watching TV and playing games, which have an adverse effect on their behavior and communication skills with parents and peers [23, 33, 34, 38]. Thus, studying how children's participation in out-of-school visual art activities varies across socioeconomic classes is crucial to understanding daily parent–child relationships and better understanding children's life chances and the reproduction of social inequality [33, 34].

### Gender role in Chinese society and extracurricular activities

Studies have witnessed that interactions in contemporary Chinese society are underpinned by the deep-rooted philosophy and teachings of Confucianism – a moral and ethical code of conduct for all human relationships that aims to cultivate an ideal and harmonious social structure [1, 19, 29, 30, 48]. One of the consequences of this societal ideology is a patriarchal culture that privileges boys due to their high rate of return for the parents, while girls are abandoned and marginalized [6, 19, 29, 30]. Li et al.’s study stipulated that the quality of both gender’s relationships with their parents is a significant determinant for their state of mental health (73); girls exhibit lower mental health than boys due to weak relationships with their parents [17, 29]. Similarly, an earlier study indicated that being in a low-SES family predicts a negative parent–child relationship for girls but not for boys [40]. This cultural ideal has driven Chinese families to invest (time and money) whatever it takes for their son to obtain high cultural, social, and human capital, whereas the ordinary provision of basic needs for a girl is more than enough [6]. Hence, parental involvement and investment in their children depend on the gender of the child, which weakens the power of SES in light of the one-child policy [12]. Fortunately, although society was restricted by the one-child policy [6], the degree of preference for boys is diminishing because of national economic development [1, 30]. Astonishingly, novel studies have shown that in affluent families, girls have a better relationship than boys with their parents [5, 7, 35]. Moreover, a study that administered a large-scale survey confirmed that being a daughter in the family considerably improves the likelihood of a positive parent–child relationship and is associated with an increased amount of time parents and children spend together [12].

On the other hand, the present study utilized visual art activities in and out of the home organized by training centers and supervised by parents. An overwhelming body of literature in China (e.g., [32, 38, 39, 48]) and internationally (e.g., [26, 27, 45, 46, 54, 55, 57]) has demonstrated that boys prefer ECAs such as sports, dancing, swimming and so on, whereas girls prefer ECAs such as painting, drawing, sculpture, music instruments, etc. Specifically, Chinese parents, primarily mothers, often prevent their children from doing something they like (e.g., going out with friends and making decisions independently) [1, 39, 44]. Additionally, these Chinese studies considered early childhood participants to assess the worth of ECAs (e.g., [31, 32, 44, 48]), which underlines the motive of this study to expand the knowledge from early adolescents’ perspectives. That makes the present study one of its kind from a different perspective. However, the study is notably unique given that a consecutive and redundant body of literature in China has accumulated that has researched only music and sports ECAs in the most developed cities in China (Beijing, Shanghai, and Hong Kong), where parents are financially able to participate in ECAs and well educated about their application [32, 38, 44, 48]. Therefore, in the current study, we aim to investigate the potential impact of family SES on a better parent–child relationship (PCR) through the serial mediating role of participation in organized visual art activities in privately owned centers (VAA1) and parental supervised visual art activities (VAA2) across genders.

### The present study

Due to swift economic development and growth, since the 1980s, China has experienced social and economic inequality and disparities that shadow its national achievement [1, 40, 58]. Unlike other countries, China has gone through a one-child policy and has a Confucian ideology that has led parents to be overprotective or practice rigid or authoritarian parenting styles, which has attracted the attention of scholars to study the interaction between children and parents [1, 6, 11, 29, 44, 58]. A preceding comparative study between American and Chinese parents underlined that US parents show a level of nonverbal, verbal, and supportive behavior to their children that surpasses that of their Chinese counterparts [11]. Similarly, an earlier comparative study among Chinese, Italian, and Costa Rica parents found that Chinese adolescents have lower maternal and parental communication and attachment than their Italian and Costa Rican equivalents [1]. Li et al. added that Confucianism caused parents to have low emotional and physical affection for their children, resulting in poor communication and parent–child relationships [1]. Thus, based on the above theoretical analyses and the existing literature, we put forward the following hypotheses [58]:

- H_1_: There is a significant visual art activity participation (VAA1 and VAA2) and quality of parent–child relationship difference between males and females.
- H_2_: The influence of VAA1 and VAA2 on the parent–child relationship is significantly different between males and females.
- H_3_: SES has a significant direct effect difference between males and females on the quality of parent–child relationships and visual art activity participation (VAA1 and VAA2).
- H_4_: VAA1 and VAA2 have significantly different serial mediating roles between males and females on the association between SES and the parent–child relationship.

### Measurements

#### SES

Due to the implication of preceding evidences and the context of the country, the socioeconomic status of our participants was measured with two common indicators: both parents’ educational levels and monthly income. Additionally, a large number of studies measure parental educational levels (father and mother) with 4- to 5-point Likert scales (e.g., [6, 12, 23]). Thus, parental occupation was not considered in the current study since China follows in the meritocracy ideology which affirms that economic goods vested in individual on the basis of their achievement, particularly academic attainment. Subsequently, the present study discusses the low SES province where society exhibits a diverse range of parental educational attainment. Accordingly, as it is presented in Table 1, parental educational level (for both fathers and mothers) was captured with the following 7-point Likert scale: 1 = did not finish elementary school (8.6% of fathers and 13.1% of mothers), 2 = completed primary school (21.4% and 19.4%, respectively), 3 = completed junior high school (36.9% and 40.0%, respectively), 4 = completed vocational high school (6.5% and 7.4%, respectively), 5 = completed regular senior high school (15.8% and 14.5%, respectively), 6 = completed junior college (15.2% and 17.4%, respectively) and 7 = completed a university undergraduate or postgraduate degree (6.8% and 8.2%, respectively). The internal consistency reliability coefficients (Cronbach's alphas) for the parental educational levels were 0.78 for the fathers and 0.75 for the mothers of our Accordingly, parental educational level (for both fathers and mothers) was captured with the following 7-point Likert scale: 1 = did not finish elementary school, 2 = completed primary school, 3 = completed junior high school, 4 = completed vocational high school, 5 = completed regular senior high school, 6 = completed junior college and 7 = completed a university undergraduate or postgraduate degree. The internal consistency reliability coefficients (Cronbach’s alphas) for the parental educational levels were 0.78 for the fathers and 0.75 for the mothers of our participants.

**Table 1:** Demographic information of the sample

	Full	Male	Female
Variables	M(SD) %	Male	Female
*Children Age*	12.6 (2.03)	12.04 (1.94)	13.16 (2.07)
*Percentage of an only child*	86.9%	79.6	94.2
*Percentage of at least one parent migrant*	33.3%	22.9	10.4
*Marital status*			
Married	80.1%	45.7	55.3
Divorce	10.5%	35.6	64.4
Remarried	7.2%	72.5	27.5
Widowed	2.2%	71.9	28.1
*Mother educational level*			
Did not finish primary school	3.1%	38.6	61.4
Primary School	6.4%	59.3	40.7
Junior high school	16.9%	45.7	54.3
Vocational High school	21.5%	62.4	37.6
Senior high school	30.2%	52.3	47.7
Undergraduate and above	21.8%	35.3	64.7
*Father Educational Level*			
Did not finish primary school	8.6%	52.8	47.2
Primary School	9.4%	67.6	36.4
Junior high school	10.0%	51.3	48.7
Vocational High school	7.4%	32.6	67.4
Senior high school	26.4%	44.9	55.1
Undergraduate and above	38.2%	62.0	38.0
*Mother monthly income*			
Under 1000RMB	18.8%	48.7	51.3
1000-3000RMB	14.0%	56.2	43.8
3000-6000RMB	20.6%	70.7	29.3
6000-9000RMB	34.4%	52.5	47.5
Over 10000RMB	12.2%	67.4	32.6
*Father Monthly Income*			
Under 1000RMB	16.1%	51.0	49.0
1000-3000RMB	10.4%	48.6	51.4
3000-6000RMB	28.5%	60.2	39.8
6000-9000RMB	21.0%	49.9	50.1
Over 10000RMB	24.0%	50.7	49.3

The distribution of parental educational levels confirms our speculation that rural Chinese parents’ educational levels are not normally distributed. The other SES indicator is monthly parental income, which was recorded from responses by each participating student’s parents or legal guardians (grandparents) on a five-point Likert scale: 1 = under 1000 RMB, 2 = 1000–3000 RMB, 3 = 3000–6000 RMB, 4 = 6000–9000 RMB, and 5 = over 9,000 RMB. The internal consistency reliability coefficients (Cronbach’s alphas) for the parental income of the fathers and mothers of our participants were 0.81 and 0.80, respectively. Finally, using principal component analysis, the parental education, and parental monthly income are integrated into a composite variable of family SES [12]. The Cronbach alpha value of the scale was 0.79, which is acceptable.

#### VAA1

The present study assessed the magnitude of children OOS visual art participation in two different instruments. The first instrument comprised of 10 items which measures children participation in organized and supervised by privately owned training centers. The items were rated on a 5-point Likert scale ranging from 1 = strongly disagree to 5 = strongly disagree. Sample items include “I like all kinds of courses related to art, “ “ I have the opportunity to discuss my artistic skill with professional artist. “ The Cronbach’s alpha coefficient value was 0.77.

#### VAA2

The second instrument that measures visual art activities contains 12 items that assesses children participation in OOS VAA2 participation supported and involved by parents in-and-out of home activities. The items were rated on a 5-point Likert scale ranging from 1 = strongly disagree to 5 = strongly disagree. Sample items were as follows: “I often go to art exhibitions or community cultural activities, “ “I draw many visual elements such as lines, shapes, spaces, and colors. “ The internal consistency reliability coefficient (Cronbach’s alpha) was 0.79.

#### Parent-child relationship

We merged two surveys used by previous studies in China to measure the relationships between parents and children [7, 20, 21]. Accordingly, after combining the relevant items and omitting identical items from both questionnaires, we had 15 items that assessed the relationship between children and parents. Some example items are “I feel a strong need to be loved unconditionally, “ “I truly need emotional support from my parents, “ and “If something goes wrong, I think I can rely on my family. “ For the sake of ensuring the trustworthiness of the present study's findings, we extend the five-point Likert scale into a seven-point Likert scale: 1 = strongly disagree, 2 = disagree, 3 = slightly disagree, 4 = neutral, 5 = slightly agree, 6 = agree, and 7 = strongly agree. The internal consistency reliability coefficient (Cronbach’s alpha) for this scale was 0.81.

### Covariates

In the current study, we controlled different covariant variables before running the models. These variables are the following; Grade (1 to 7), age (7 to 14), residence (rural = 1, urban = 0), parental work status (migrant = 1, non-migrant = 0), and race (1 = Han, 0 = Minority).

## Method

### Participants

A large number of studies China literature reviewed in the present study extract their dataset from the national panel survey data conducted in 2013/14 that has a weak implication for the current social policy, practical and theoretical contribution. (e.g., [6, 12, 17, 36]). Therefore, after obtaining the first author's university (Shenzhen University) ethical committee approval, we randomly recruited 1890 primary school students sampled in Y Province in southwestern China. Voluntary participants’ informed consent was obtained from each student's parents before they took part in the study. Later, with the help of school teachers and administrations, eligible participants were required to respond each questions in the survey, which consisted of the study aimed to measure. Subsequently, we ought to omit 266 students' invalid responses; the study analyzed 1624 children's responses. A primary school in China represents children aged 7 to 14 attending grade levels from 1 to 6. In our sample, the average age of the primary children was (M = 11.6, SD = 2.03), and 52.5% were girls (See Table 1).

### Analysis procedure

We administered descriptive and correlation analysis across male and female participants using SPSS 22.0 to provide preliminary findings in the current study. Then, AMOS 21.0 software to perform the serial mediation role of VAA1 and VAA2 on the association of SES and parent–child relationship using structural equation model (SEM). All continuous variables run in the models were standardized and employed bootstrapping test among male and female mediation models to estimate bias-corrected confidence interval with 5000 bootstraps sample for the models' direct, indirect, and total effect. Thus, the 95% confidence interval, which consists of zero considered as an insignificant effect and vice versa.

## Results

### Descriptive analysis

The finding from independent t test analysis shows that gender has a significant effect on the likelihood of children's participation in VAA1 (F (1) = 21.83, *p* < 0.001, η^2^ = 0.13) and VAA2 (F (1) = 29.86, *p* < 0.001, η^2^ = 0.18) visual art activities. Astonishingly, the mean comparison result indicates that the parent–child relationship means the difference between males and females is nearly zero (F (1) = 0.096, *p* = 0.756, η^2^ = 0.001). Alternatively, the t test finding from Table 2 stipulates that being a son or daughter does not influence the quality of the parent–child relationship. Hence, this finding partially supports our first hypothesis that posits the intensive participation of female adolescents in visual art activities compared to their male counterparts but rejects the quality of parent–child relationship differences between the two groups.

**Table 2:** Descriptive analysis and comparison of means across gender

	VAA1			VAA2			PCR		
	M	SD	F	M	SD	F	M	SD	F
*Gender*			21.83***			29.86***			.096
Male	2.9442	.79193		3.03	.896		3.74	.912	
Female	3.1212	.73448		3.26	.839		3.73	.874	

^*^p < 0.05, **p < 0.01, ***p < 0.001. *VA1* Organized Visual art activities by private training centers, *VAA2* in-and-out of home visual art activities supervised by parents, *PCR* Parent–child relationship

Table 3 presents the correlation analysis of the primary variables studied in the present paper across male and female samples. As we expected, the bivariate correlation result demonstrates that for both groups, maternal educational (r_boy_ = 0.621, *p* < 0.001, r_girl_ = 0.651, *p* < 0.001) and income (r_boy_ = 0.751, *p* < 0.001, r_girl_ = 0.621, *p* < 0.001) have a more robust association with a positive parent–child relationship than parental educational attainment (r_boy_ = 0.452, *p* < 0.001, r_girl_ = 0.525, *p* < 0.001) and income (r_boy_ = 0.321, *p* < 0.001, r_girl_ = 0.470, *p* < 0.001). Likewise, the Pearson correlation result found that maternal educational level (r_boy_ = 0.792, *p* < 0.001, r_girl_ = 0.749, *p* < 0.001; r_boy_ = 0.741, *p* < 0.001, r_girl_ = 0.749, *p* < 0.001) and income level (r_boy_ = 0.650, *p* < 0.001, r_girl_ = 0.769, *p* < 0.001; r_boy_ = 0.701, *P* < 0.001, r_girl_ = 0.785, *p* < 0.001) significantly and vigorously determined male and female student participation in VAA1 and VAA2, respectively, compared with parental educational and income level. Most importantly, the correlation result elucidates that male and female participation in VAA2 (r_boy_ = 0.783, *p* < 0.001, r_girl_ = 0.883, *p* < 0.001) has a more substantial impact on the quality of the parent–child relationship than VAA1 (r_boy_ = 0.606, *p* < 0.001, r_girl_ = 0.627, *p* < 0.001).

**Table 3:** Pearson correlation among operating variables

	M	SD	1	2	3	4	5	6	7	M	SD
FE	3.27	1.50		.652	.543	.224	.525	.532	.544	3.37	1.44
ME	3.13	1.52	.652		.457	.657	.714	.769	.749	3.13	1.47
FI	2.54	1.06	.243	.157		.524	.470	.440	.582	2.39	.972
MI	2.08	.954	.224	.457	.524		.621	.650	.785	2.06	.909
PCR	3.74	.912	.452	.321	.597	.752		.627	.883	3.7286	.874
VAA1	2.94	.792	.432	.649	.440	.650	.606		.692	3.1212	.735
VAA2	3.03	.896	.344	.701	.482	.789	.783	.792		3.2616	.839

*FE* Father Education, *ME* mother education, *FI* Father Income, *MI* Mother Income, all correlation values are significant *P* < 0.001

### The mediating role of VAA1 and VAA2 between SES and the parent–child relationship

**Fig. 1:**
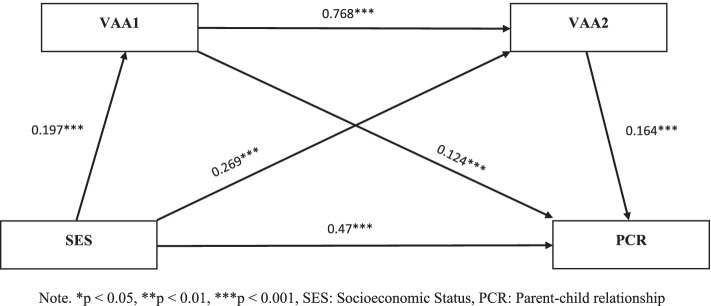
The multiple mediation model of socioeconomic status on the parent–child relationship among total samples (*N* = 1624). Note. *p < 0.05, **p < 0.01, ***p < 0.001, SES: Socioeconomic Status, PCR: Parent–child relationship

**Fig. 2:**
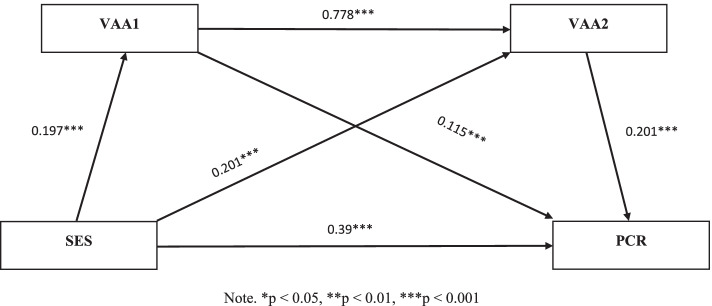
The multiple mediation model of socioeconomic status on the parent–child relationship among the male sample (*N* = 772). Note. *p < 0.05, **p < 0.01, ***p < 0.001

**Fig. 3:**
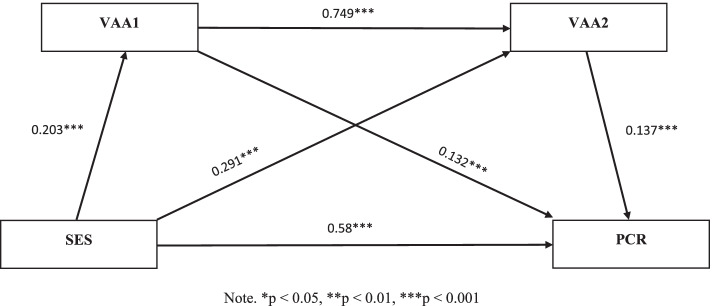
The multiple mediation model of socioeconomic status on the parent–child relationship among the female sample (*N* = 852). Note. *p < 0.05, **p < 0.01, ***p < 0.001

Table 4 and Fig. 1 show each path's direct and indirect effects regressed in our multigroup serial mediation analysis with 5000 sample bootstrapping estimations. According to the total sample SEM, SES has a significant direct effect on the parent–child relationship (β = 0.47, *p* < 0.001), participation in VAA1 (β = 0.197, *p* < 0.001) and VAA2 (β = 0.269, *p* < 0.001). Moreover, the path analysis confirms that the quality of the parent–child relationship is more highly determined by student participation in VAA2 (β = 0.124, *P* < 0.01) than VAA1 (β = 0.164, *p* < 0.001). Most interestingly, Table 4 and Figs. 1, 2 and 3 affirm that except for participation in VAA2 (β_girl_ = 0.137, *p* < 0.001; β_boys_ = 0.201, *p* < 0.001), the multigroup analysis claimed that females’ positive parent–child relationship is notably predicted by SES (β_girl_ = 0.58, *p* < 0.001; β_boys_ = 0.39, *p* < 0.001) and participation in VAA1 (β_girl_ = 0.132, *p* < 0.05; β_boys_ = 0.115, *p* < 0.05) compared to males, which confirms the second hypothesis of the current study. In addition, the likelihood of female student participation in VAA1 (β_girl_ = 0.203, *p* < 0.001; β_boys_ = 0.197, *p* < 0.001) and VAA2 (β_girl_ = 0.291, *p* < 0.001; β_boys_ = 0.201, *p* < 0.01) is more strongly dependent than male students on the SES of parents. Altogether, these findings support our third hypothesis that the power of SES in determining the quality of the parent–child relationship and tendency of participation in VAA1 and VAA2 is different in male and female groups.

**Table 4:** Direct and indirect effect SES on the parent–child relationship

					Multi-group Model							
	Full Model (*N* = 1624)				Male (*N* = 772)				Female (*N* = 852)			
Paths	β	SE	95% CI		β	SE	95% CI		β	SE	95% CI	
SES-PCR	0.47***	0.001	[0.06, 0.08]		0.39***	0.067	[0.15, 0.39]		0.58***	0.012	[0.21, 0.57]	
SES-VAA1-PCR	0.06**	0.004	[0.02, 0.05]		0.04**	0.052	[0.37,0.65]		0.08**	0.036	[0.17, 0.22]	
SES-VAA2-PCR	0.08***	0.002	[0.01, 0.06]		0.08**	0.072	[0.44, 0.53]		0.08***	0.041	[0.16, 0.30]	
SES-VAA1-VAA2-PCR	0.01***	0.052	[0.024, 0.051]		0.01***	0.104	[0.35, 0.46]		0.01***	0.081	[0.41, 0.48]	
**Model Fit**	***x***^**2**^**/*df***	**CFI**	**GFI**	**RMSEA**	***x***^**2**^**/*df***	**CFI**	**GFI**	**RMSEA**	***x***^**2**^**/*df***	**CFI**	**GFI**	**RMSEA**
	51.47***	0.947	.997	0.048	49.72***	0.965	.978	0.040	53.71***	0.970	.989	0.045

^*^p < 0.05, **p < 0.01, ***p < 0.001

Furthermore, after adding the mediating variable, the result revealed that SES has a significant indirect effect on the parent–child relationship through VAA1 (β = 0.06, *p* < 0.01) and VAA2 (β = 0.08, *p* < 0.001). For the male and female groups, the mediation result indicates that SES has a stronger indirect effect on the parent–child relationship through a mediating role of VAA1 (β_girl_ = 0.08, *p* < 0.01; β_boys_ = 0.04, *p* < 0.01) for female than male samples of the regression. However, the mediating effect of VVA2 between SES and the parent–child relationship in the female (β = 0.08, *p* < 0.001) and male (β = 0.08, *p* < 0.01) models is equal, although female gender is highly significant. These findings verify that VAA1 and VAA2 have a significant serial mediating role in SES and the parent–child relationship. The exclusive finding of the serial mediation test is that VAA1 and VAA2 have equal effect and significant value in a serially mediating role between SES and parent–child relationship in total (β = 0.01, *p* < 0.001), male (β = 0.001, *p* < 0.001), and female (β = 0.001, *p* < 0.001) samples. Although the finding affirms that VAA1 and VAA2 have a serial mediating role between SES and the parent–child relationship in the multigroup setting, the finding refutes our last hypothesis.

## Discussion

The paramount aim of the current study was to examine the potential influence of SES on the quality of the parent–child relationship through the serial mediating role of children's participation in VAA1 and VAA2. The primary motive of the study emerged from the lack of literature about the application of visual art activities in the noncognitive aspects of children, the need to understand the role of gender in contemporary Chinese society in accessing cultural capital, and most importantly, we sought to put forward relevant implications for the recent educational policy reform by the Chinese government, which assists children to use their out-of-school or afterschool time to prioritize extracurricular activities that fortify positive parent–child relationships, which are known to be at the core of children’s future development. Thus, our study intended to scrutinize the relationship between SES and the parent–child relationship via the serial mediation of participation in visual art activities organized by private art centers (VAA1) and visual art activities in and out of home supervised by parents (VAA2).

The preliminary finding of the study illustrates that females have a higher likelihood of participating in VAA1 and VAA2 than males; as we expected, this finding supports a large volume of literature that claim that female children and adolescents prefer to spend their leisure time on visual art activities, while male counterparts favor engaging in other ECAs such as sports, dance or swimming [26, 27, 32, 38, 39, 45, 46, 48, 54, 55, 57]. In contrast, our T test finding shows that the gender of the child does not determine a positive or negative parent–child relationship, which made us partially accept our first hypothesis. This is a unique finding that differs from preceding and recent national evidence, which stipulates that females have levels that exceed [5, 7, 12, 35] or are lower than [17, 29, 40] those of the quality of the parent–child relationship of their male equivalents. The possible reason for this finding is that from our total sample, 86.9% are only children for their parents; thus, as a contemporary study noted, only daughters have a higher possibility of maintaining a quality relationship with their parents [6], whereas being a son carries a privilege as an only child or in a family with siblings [19, 29, 30].

According to the study's primary objective, we test a serial meditating role of VAA1 and VAA2 between the association of SES and the parent–child relationship. An emerging body of studies has shown the significant mediating role of the parent–child relationship between several explanatory and dependent variables, which explain different dimensions of children's development [7, 13, 17, 35]. Fortunately, the present study sheds light on assessing possible variables that mediate the parent–child relationship that broaden and expand the parent–child relationship with other determiners. The primary relation we identified extended from the family investment model in light of our second hypothesis was the effect of VAA1 and VAA2 on the parent–child relationship among males and females. Predictably, both forms of visual art activities predict a positive parent–child relationship across both genders.

Notwithstanding VAA1 and VAA2 exhibit to ensure a solid parent–child relationship, shared visual art activities supervised by parents, performed in-home and out of home by visiting art museums, exhibitions, or galleries, make the parent and child bond more potent than before. This finding confirms our second hypothesis, given that participation in VAA2 requires both financial and time investment of parents, which creates a sense of belongingness, safety and confidence among both genders of children [6, 7, 23]. This finding explains that children participate in sole visual art activities; it is better for parents to companion their children in different shared visual art activities that offer transparent time between parents and children that allows parents to understand their children’s perspectives and react to them.

Drawing on Bronfenbrenner’s ecological theory, the family is labeled as a microsystem described by multilevel variables. According to the present study, the theory is extended to elucidate the association between family SES, children's VAA1 and VAA2 participation, and the parent–child relationship. The finding of the structural equation model demonstrates that the quality of the parent–child relationship and participation of males and females in VAA1 and VAA2 are determined by their parents’ SES. The path analysis found that SES determines children's participation in VAA1 and VAA2; the effect was notably strong in the female model. In line with our third hypothesis, affluent children obtain a healthy living environment that secures a positive parent–child relationship [1, 5, 12, 13, 17, 29, 35, 36, 40], given that parents of a high social class have a clear understanding of the consequences of fostering and maintaining smooth communication and relationships with their children, which is not biased by their child’s gender [7, 35, 38, 39]. Furthermore, our finding is consistent with previous studies, which indicated that maternal educational attainment has a robust influence on the positive parent–child relationship [35, 40], and has an influence on children participating in extracurricular activities in their leisure time [33, 45].

Regarding the separate mediating role of VAA1 and VAA2 between SES and parent–child relationship, VAA1 has a higher mediating effect for female models than male groups and the total sample. A possible explanation for this finding is that female adolescents are passionate about visual art activities, which makes them request that their parents send them to training centers that foster their art skills and creativity [32, 38, 39, 55, 57]. Thus, involving those activities in more organized training with trained teachers made them love, appreciate, and respect their parents, potentially strengthening the parent–child relationship. In contrast, the mediating effect of VAA2 on the relationship between SES and the parent–child relationship is equal across male and female group models. This explains that, unlike VAA1, although visual art activity is less preferred among males, participating in supervised visual art activities that require an intensive shared activity of children and parents equally accounts for the association of the parent–child relationship with SES among males and female models. In addition, this finding confirms that traditional Chinese parenting is diminishing, which elucidates that the gender of a child considerably determines the intensity of parental devotion in terms of money and time (e.g., [1, 6, 19, 29, 30]). Likewise, the potential explanation for the present study finding is that male adolescents benefit equally to their female counterparts from visual art activities in light of their involvement in a shared visual art activity that requires intensive guidance, support, and supervision from parents. In contrast to our fourth hypothesis, VAA1 and VAA2 have a significant and equal serial mediating role in the association between SES and the parent–child relationship among the male and female models. Fortunately, the uniform serial mediating role of VAA1 and VAA2 in the multigroup reveals that both male and female children can equally restore their relationship with their parents by having substantial participation in both VAA1 and VAA2. Therefore, as the findings of the serial model demonstrate, unlike a large body of evidence, male adolescents could maintain a strong parent–child relationship through visual art activities as long as they obtain a considerable asset in participating in VAA1 and VAA1. However, it must be noted that only affluent children and adolescents would be able to enroll in private art classes and be accompanied by their parents [32, 38].

Due to the four limitations of the current study, the findings need to be interpreted and understood under the following caveats. First, since the study approaches the problem in a cross-sectional research design, we cannot claim a causal relationship among the studied variables. Thus, this study highly stimulates scholars to explore such relationships in longitudinal or experimental research designs to claim cause-effect relationships. Second, the study would be more robust if we could collect data from parents in a survey or interview to triangulate or strengthen our findings. Third, the study was conducted in Y province located in the southwestern part of China, so the generalization radius of the present study is limited to the studied province. Fourth, the current study examines the mediating role of visual art activities as one of the ECAs mediating the relationship of SES and the parent–child relationship, so the study's finding only concerns the application of visual art activities.

## Conclusions

The first empirical study examines the potential mediating role of visual art activities on the association between SES and parent–child relationships in Y province of southwestern China. In addition, unlike previous national and international studies, the present study measured visual art activities in two dimensions for a robust finding that forward feasible policy and practical implications. The primary findings are the following: (a) Although in contemporary China, females participate highly in visual art activities (VAA1 and VAA2), the quality of the parent–child relationship is equal among males and females. (b) Affluent children (male or female) have a high possibility of participating in any visual art activities (VAA1 and VAA2). (c) There were significant differences in the serial mediation role of VAA1 and VAA2 between boys’ and girls’ samples, as SES predicts the quality of the parent–child relationship.

To the best of our knowledge, this study is the first to examine such serial mediating roles in the association of crucial explanatory and predicted variables. The findings of the present study have essential policy and practical implications. The first policy implication the study suggests is that as the government stimulates parents to involve their children in several ECAs, feasible considerations must be taken to attract economically disadvantaged children by establishing community service centers where low-SES children and parents participate in different ECAs at a low cost. In addition to out-of-school ECAs, schools need to design a curriculum to add relevant activities such as visual art, music, and sports, and the government has to support and monitor the implementation. Regarding the study's practical implications, parents ought to send their children to training centers, but they are also suggested to accompany them to activities to explore their children's interests, wishes, and plans. Children feel happy, secure, and loved when parents suggest, compliment, and support their work, so parents should devote more time than money to their children's development. Moreover, a different awareness must be strengthened to eliminate gender preference in all parents through school, social and entertainment gatherings. Ultimately, we highly encourage future studies in other provinces and other countries to strengthen or argue the possible finding of the present study that male adolescents could benefit from visual art activities, as long as studies explicitly define the nature of the activities they are involved in. Thus, future studies must consider measuring ECAs or visual art activities in depth for robust implications that would contribute to children’s well-being and development.

## Data Availability

The datasets used and/or analyzed during the current study are available from the corresponding author on reasonable request.
